# Artificial intelligence application in the diagnosis and treatment of bladder cancer: advance, challenges, and opportunities

**DOI:** 10.3389/fonc.2024.1487676

**Published:** 2024-11-07

**Authors:** Xiaoyu Ma, Qiuchen Zhang, Lvqi He, Xinyang Liu, Yang Xiao, Jingwen Hu, Shengjie Cai, Hongzhou Cai, Bin Yu

**Affiliations:** ^1^ Department of Urology, Jiangsu Cancer Hospital & The Affiliated Cancer Hospital of Nanjing Medical University & Jiangsu Institute of Cancer Research, Nanjing, Jiangsu, China; ^2^ Department of Radiology, The Fourth School of Clinical Medicine, Nanjing Medical University, Nanjing, Jiangsu, China; ^3^ School of Public Health, Southern Medical University, Guangzhou, Guangdong, China; ^4^ The Third Clinical Medical College, Nanjing University of Chinese Medicine, Nanjing, Jiangsu, China

**Keywords:** bladder cancer, artificial intelligence, deep learning, machine learning, radiation therapy

## Abstract

Bladder cancer (BC) is a serious and common malignant tumor of the urinary system. Accurate and convenient diagnosis and treatment of BC is a major challenge for the medical community. Due to the limited medical resources, the existing diagnosis and treatment protocols for BC without the assistance of artificial intelligence (AI) still have certain shortcomings. In recent years, with the development of AI technologies such as deep learning and machine learning, the maturity of AI has made it more and more applied to the medical field, including improving the speed and accuracy of BC diagnosis and providing more powerful treatment options and recommendations related to prognosis. Advances in medical imaging technology and molecular-level research have also contributed to the further development of such AI applications. However, due to differences in the sources of training information and algorithm design issues, there is still room for improvement in terms of accuracy and transparency for the broader use of AI in clinical practice. With the popularization of digitization of clinical information and the proposal of new algorithms, artificial intelligence is expected to learn more effectively and analyze similar cases more accurately and reliably, promoting the development of precision medicine, reducing resource consumption, and speeding up diagnosis and treatment. This review focuses on the application of artificial intelligence in the diagnosis and treatment of BC, points out some of the challenges it faces, and looks forward to its future development.

## 1 Introduction

Bladder cancer (BC) is the fourth most common type of cancer among men and the second most common urological reproductive system tumor. The American Cancer Society projects that in 2024, a total of 83,190 people will be diagnosed with BC, and 16,840 will die as a result (1). More than 95% of BCs are uroepithelial carcinomas. At diagnosis, approximately 75% of cases will be non-muscle invasive BC (NMIBC), with the remainder being muscle-invasive BC (MIBC) or metastatic disease (2, 3). BC has one of the highest lifetime costs of treatment per patient disease and represents a major challenge for oncology given its wide range of disease risks, management options, and complications (4).

Artificial intelligence (AI) is a machine’s ability to mimic human intelligence to perform tasks involving decision-making and problem-solving. With the rapid development of AI in recent years, its use in healthcare has become increasingly widespread. The more common AI techniques in clinical medicine include deep learning (DL) and machine learning (ML). ML refers to the ability of a machine to develop algorithms that make predictions about data based on trends and patterns in previous data (5). DL is a subset of ML that involves hierarchical and model learning through neural networks and allows predictions to be made in unstructured environments (6). AI will play an essential role in addressing the unmet needs of a BC visit, including imaging tests, pathologic diagnosis, molecular biomarkers, risk stratification, treatment assessment, and outcome prediction, among many others.

The goal of this review is to review existing technologies and current research and discuss the role that AI currently plays in the BC diagnostic and treatment process.

## 2 AI in the diagnosis of bladder cancer

### 2.1 Cystoscopy

Cystoscopy is a standard tool for diagnosing and monitoring BC and plays an essential role in diagnosing and treating BC. However, it is reported that the misdiagnosis rate of white light cystoscopy is up to 30% (7), and the sensitivity and specificity of diagnosis under white light imaging (WLI) are 60% and 70%, respectively (8). Given that AI can recognize pixel-level features that cannot be detected by the human eye and has a solid autonomous learning ability, it has the potential to be applied in the medical diagnosis field and improve the accuracy rate. **Table 1** describe some of the applications of AI in cystoscopy.

**Table 1:** Artificial intelligence in cystoscopy.

Year of Study	Model	Training set	Test set and validation set	Result	Reference
2022	Pretrained pyramid-scheme parsing network fine-tuned with cystoscopy dataset	5,013 tumor images and 46,054 normal images	1,261 tumor images and 11,124 normal images (internal validation); 647 tumor images and 5,105 normal images from1,427 patients (external validation)	Accuracy = 0.939, 95% CI = 0.902 to 0.964; sensitivity = 0.954, 95% CI = 0.902 to 0.983	(9)
2020	Pretrained GoogleNet with fine-tuned cystoscopy dataset	344 tumor images and 1,336 normal images	87 tumor images and 355 normal images (test)	The AUC value was 0.98, the sensitivity was 0.897, and the specificity was 0.94	(10)
2021	A deep learning network-assisted bladder tumor recognition under cystoscopy based on Caffe deep learning framework and EasyDL platform	734 tumor images from 72 patients and 1,002 normal images from 103 patients	4 untrained images of cystoscopy (validation)	The accuracy rate of the neural network to recognize the bladder cancer based on Caffe framework was 0.829, and the data on the EasyDL platform were 0.969	(11)
2019	CystoNet, an image analysis platform based on CNNs for automatic BC detection	95 patients	5 patients (test) and 54 patients (validation)	Per-frame sensitivity = 0.909, 95% CI = 0.903 to 0.916; specificity = 0.986, 95% CI = 0.985 to 0.988	(12)
2022	Feature fusion, transfer learning and CapsNets	90% of the digital atlas (256 normal frames from 12 patients, 144 T1 tumor frames from 6 patients and 53 T1 tumor frames from 5 patients)		The accuracy rate increased from 0.946 to 0.969	(13)
2018	Deep CNN models (ResNet50, VGG-19, VGG-16, InceptionV3, and Xception)	60% of the digital atlas (479 images from 44 cystoscopy results)	10% of the digital atlas (validation) and 30% of the digital atlas (test)	The Classification Accuracy of the Xception-based model was 0.9952, followed by models that were based on ResNet50 (0.9948) and the harmonic-series concept (0.9945)	(14)
2020	Dataset Description, Laplacian edge detector, Image Resizing, MLP with Laplacian of an image	1,500 tumor images and 500 normal images	497 tumor images and 486 normal images (test)	The AUC were 0.99 (50×50 input images), 0.99 (100×100 input images), 0.96 (30×30 input images) and 0.92 (200×200 input images) respectively.	(15)
2021	three classic deep learning networks (LeNet, AlexNet, and GoogLeNet) and the EasyDL platform	80% of the digital atlas (1,200 tumor images from 224 patients and 1,150 normal images from 221 patients)	20% of the digital atlas (1,200 tumor images from 224 patients and 1,150 normal images from 221 patients)	The accuracy was 0.969 of EasyDL, 0.9254 of GoogLeNet, 0.8336 of the neural network and 0.8409 (p > 0.05) of medical expert	(16)
2023	VISIOCYT1		391 patients (170 patients with BC and 149 patients without BC)	Overall sensitivity = 0.809, 95% CI = 0.739 to 0.864; specificity = 0.618, 95% CI 0.534 to 0.695; sensitivity = 0.937, 95% CI = 0.86 to 0.973 in high-grade tumors and 0.667, 95% CI 0.552 to 0.765% in low-grade tumors	(17)

BC, bladder cancer; CNN, convolutional neural network; CI, confidence interval; AUC, area under curve.

Wu et al. (9) built the Cystoscopy AI Diagnostic System (CAIDS) framework using a pyramid scene parsing network (PSPNet) trained on ImageNet and the training set. To compare the performance of CAIDS and urologists in identifying BC, 69,204 images of 10,729 patients were used to develop CAIDS, and finally a subgroup of 260 images in complex lesions was used for the test. In the comparisons, the CAIDS showed high accuracy and sensitivity (accuracy = 0.939, 95% confidence interval (CI) = 0.902 to 0.964; sensitivity = 0.954, 95% CI = 0.902 to 0.983). With a latency of 12 seconds, the CAIDS is more accurate and quicker than expert urologists. Ikeda et al. (10) jointly developed a convolutional neural network (CNN) -based tumor classifier using 431 tumor lesion images obtained from 124 bladder resections performed on 109 patients, plus 1671 standard tissue images. The test results showed that the area under the curve (AUC) value was 0.98, the sensitivity was 89.7%, and the specificity was 94.0%. Du et al. (11) used the Caffe DL framework and the EasyDL platform for model construction and training. Cystoscopy was performed on 175 patients, and 1002 photos of normal bladder tissue and 734 photos of bladder tumors were taken. The training results showed that the accuracy rate of neural networks based on the Caffe framework in identifying BC is 82.9%, and the data on the EasyDL platform is 96.9%. Shkolyar et al. (12) aimed to develop a DL algorithm for enhancing the detection of BC by cystoscopy. Shkolyar et al. constructed an image analysis platform named CystoNet based on CNNs for automatic BC detection. The algorithm was trained using the development dataset of 95 patients and tested with 5 patients. As the per-frame sensitivity and specificity were 90.9% (95% CI, 0.903 to 0.916) and 98.6% (95% CI, 0.985 to 0.988), DL-enhanced cystoscopy may improve tumor localization, intraoperative navigation, and surgical resection of BC. Freitas et al. (13) confronted two methods for classifying bladder lesions shown in white light cystoscopy images: the classic method, namely the feature-feeding pattern recognition system based on manual design, and the method based on modern DL. They found that with more robust models, elaborately hand-designed features can perform similarly to traditional DL-based models and deep capsule networks, improving accuracy from 94.6% to 96.9% and making it more conducive to clinical applications.

Eminaga et al. (14) used neural network models such as ResNet50, VGG - 19, VGG - 16, InceptionV3, and Xception to classify cystoscopic images, and developed two convolutional neural network architectures. They trained, validated, and evaluated the models by randomly generating a training set (60%), a validation set (10%), and a test set (30%). The results showed that the Xception model had the highest F1 score (99.52%), followed by the ResNet50 model (99.48%) and the model based on the concept of harmonic sequence (99.45%). The introduced models could accurately identify malignant urological results and distinguish between interstitial cystitis, other types of cystitis, and carcinoma *in situ*. Lorencin et al. (15) used MLP with different activation functions and optimizers to train and test bladder cancer images of various sizes, obtained the AUC value and training time through ROC analysis to determine the optimal hidden layer design and image size. Except for the 30×30 images, the maximum AUC can be achieved using MLP models with medium complexity. The highest AUC values can be obtained with 50×50 and 100×100 input images, which are 0.99 and 0.99, respectively; the AUC values of 30×30 and 200×200 input images are 0.96 and 0.92, respectively. Lorencin et al. (18) used the two widely used CNN architectures, AlexNet and VGG16, and fine-tuned them to adapt to grayscale images to study the impact of GAN-based image data augmentation on the urinary bladder cancer diagnosis system. The results show that GAN-based augmentation influences the classification and generalization performances of CNNs. For AlexNet, this approach improves the average and median performances, makes the network behavior more stable, and significantly enhances the generalization performance. Yang et al. (16) collected 1200 cystoscopic cancer images from 224 patients with bladder cancer and 1150 cystoscopic images from 221 patients without bladder cancer and used three classic deep learning networks (LeNet, AlexNet, and GoogLeNet) and the EasyDL platform to build the model. The accuracy rates of all three models were relatively high, and there was no overfitting phenomenon. GoogLeNet had the highest accuracy rate, and EasyDL had the highest F1 score in the V2 round, with an accuracy rate of 96.9% then. VISIOCYT1 (17) was a French prospective clinical trial conducted in 14 centers. The main objective is to evaluate the diagnostic performance of the VisioCyt test, including sensitivity and specificity, and to assess the sensitivity of tumor grading, T staging, and the trial groups. The overall sensitivity is 80.9%, and the specificity is 61.8%; the sensitivity in high-grade tumors is 93.7%, and in low-grade tumors, it is 66.7%.

Cystoscopy generally has problems such as high cost, invasiveness, and dependence on the operator. For young doctors, it is challenging to interpret the results of cystoscopy, and it relies on the examiner’s skills. Artificial intelligence can be used to objectively evaluate cystoscopy images, with high sensitivity and specificity, and can assist in diagnostic classification. For example, CystoNet, convolutional neural networks, and deep learning models can be applied in diagnostic support, image classification, tumor detection, and performance evaluation, which is expected to improve the accuracy of bladder cancer diagnosis and treatment, reduce the examination time, and overcome the limitations of human expert opinions. However, the algorithm is sensitive to the amount of input data, and collecting medical data is challenging, usually requiring data augmentation. High-quality input data and image preprocessing are crucial for developing a powerful diagnostic classification model. In the future, AI-assisted cystoscopy should be integrated into clinical routines and may be extended to other clinical endoscopic applications. More cystoscopy data and a more robust network structure are needed to explore the highest accuracy of convolutional neural networks in bladder tumor recognition.

### 2.2 Computed tomography (CT)

In recent years, AI has made some achievements in CT imaging detection, which makes people realize the great prospect of ML and DL models based on CT images in the pathological grading and invasive evaluation of BC (19). ML and DL techniques use CNNs to discover complex structures and patterns in CT images (20). ML and DL carry out image recognition diagnosis and personalized medicine to improve diagnostic accuracy and speed up the clinical diagnosis and treatment. Garapati et al. (21) used an objective computer-aided system to assess the BC stage. The data set of 84 BC lesions indicated promise as a classification tool for stratifying BC into two staging categories. The ML algorithm prediction model developed in their study demonstrated the feasibility of ML for predicting the extent of BC infiltration. Zhang et al. (22) developed a DL model based on CT images for predicting the muscle invasion status of BC. The model had good predictive ability for the preoperative muscle invasion status of BC.

AI can enhance the clinical value of CT image reports. Cha et al. (23) compared deep learning convolution neural networks (DL-CNN) with auto-initialized cascaded level sets (AI-CALS). Their results showed that DL-CNN can produce accurate BC segmentation. Gresser et al. (24) compared radio histological analyses of manual and automated lymph node (LN) segmentation for detecting LN metastases in BC in 1354 BC patients undergoing radical cystectomy combined with LN dissection. The study found that models based on manual LN segmentation were superior to fully automated methods. There is currently less research in this area, and further research is needed on the role of AI in this area.

Moreover, CT imaging histology-based modeling in objective clinical benefits performs well. Borrelli et al. (25) used CT-based AI-powered software to evaluate objective clinical benefits in 2021. The outcomes of clinical benefit in 66 patients with BC showed a positive association with the observed anthropometric features.

### 2.3 Magnetic resonance imaging (MRI)

Combining AI and MRI, especially in BC staging using multiparametric MRI (mpMRI), gives AI a powerful ability to identify cancer-invasive muscle and pathological grading. Predicting the degree of muscle infiltration of BC by DL-CNN is a helpful method for the diagnosis and treatment of BC.

The MRI-based AI model can improve the detection rate of BC (14). Zhang et al. (26) described textural features of different grades of BC from diffusion-weighted images and apparent diffusion coefficient maps. The selection of optimal texture features improved the ability to differentiate BC from normal bladder wall tissue and facilitate image-based BC grading preoperatively. The MRI-based AI model can also save the cost of the doctors’ time. Ye et al. (27) used semi-automated and manual segmentation results to differentiate between NMIBC and MIBC in radiomics analyses. The outcomes showed they had a similar diagnostic performance, but the semi-automatic segmentation method was less time-consuming and required fewer manual interventions.

Compared to CT, MRI has better soft tissue resolution and can be used to assess the extent of BC infiltration (28). Xu et al. (29) studied T2 weighted MR images (T2WI) of 68 patients with BC. The outcome showed that T2WI and its higher-order derivative maps could reflect muscle invasiveness in BC. Xu et al. (30) evaluated the discriminatory performance of preoperative mpMRI radiomics features for the precise identification of NMIBC and MIBC in the follow-up study, demonstrating the advantages of mpMRI. Li et al. (31) constructed a T2WI-based radiomics model, a single-task DL model, and a multi-task DL model and predicted MIBC status. The study showed that multi-task DL paid more attention to the diseased tissue area and was more reliable for the clinical references. AI can improve the sensitivity and specificity of tumor detection by analyzing MRI images of BC patients and by analyzing different texture features in response to various tissue types of BC lesions. Meanwhile, AI-assisted MRI diagnosis can more accurately assess the degree of muscle infiltration of BC and provide an important basis for developing clinical treatment plans based on whether it is MIBC.

### 2.4 Ultrasound

Ultrasound has high sensitivity and specificity in diagnosing BC (32). Moreover, as ultrasound is a non-invasive, recognized, and cost-effective diagnostic technique, it has advantages in some cases compared with the invasive operation of cystoscopy (33). The application of AI in ultrasound for BC is helpful. It can improve the diagnostic accuracy. The AI algorithm can conduct a more detailed and precise analysis of the ultrasound images, helping to identify the subtle lesions in the early stage of BC, reduce the subjectivity and error of human judgment, and provide doctors with a more objective and consistent diagnostic basis. Gao et al. (34) established a radiomics model based on ultrasound images. A total of 157 patients were included for analysis; 104 cases were used to evaluate the tumor stage, and 143 cases were used to assess the pathological grade. For the tumor stage prediction model, the AUC, sensitivity, and specificity in the training set were 0.94, 0.77, and 0.98, respectively; the AUC, sensitivity, and specificity in the validation set were 0.84, 0.77, and 0.89, respectively. For the pathological grade prediction model, the AUC, sensitivity, and specificity in the training set were 0.84, 0.80, and 0.78, respectively; the AUC, sensitivity, and specificity in the validation set were 0.75, 0.87, and 0.60, respectively. All models showed good predictive ability, which was helpful for effective risk stratification for patients. It is expected to provide a non-invasive, economical, reliable, and effective preoperative prediction tool for tumor staging and pathological grading, helping doctors make accurate diagnoses, choose appropriate individualized treatment plans, and improve the prognosis of patients.

### 2.5 Urine cytology

Urine cytology (35) is a method of observing various cells in the urine sediment with a microscope and evaluating their significance. It is mainly used to detect malignant cancers in the urinary system and is the first non-invasive diagnostic method for BC that is widely used in clinical practice. **Table 2** describes the application of various AI models in Urine cytology.

**Table 2:** Application of artificial intelligence in urine cytology.

Year of Study	Model	Training set	Test set	Result	Reference
2023	AutoParis-X, an improved semiautonomous urine cytology assessment tool	47 urine specimens (total: 1303 urine specimens from 140 patients)	1252 urine specimens (total: 1303 urine specimens from 140 patients)	AutoParis-X can accurately determine urothelial cell atypia and aggregate a wide variety of cell-related and cluster-related information across a slide to yield an atypia burden score	(36)
2023	VisioCyt, an *in-vitro* diagnostic medical device		391 patients (170 patients with BC and 149 patients without BC)	Sensitivity = 0.809, 95% CI = 0.739 to 0.864; specificity = 0.618, 95% CI = 0.534 to 0.695	(17)
2024	A fully automated artificial intelligence system used DL to predict histologically advanced urothelial carcinoma from digitized urine cytology slides	181 consecutive urine cytology slides	39 consecutive urine cytology slides (cell-level) and 315 consecutive urine cytology slides (slide-level)	The sensitivity of histological HGUC to predict AI was 63%, which was superior to the sensitivity of cytology by pathologists (46%) (p = 0.0037).	(37)
2024	Precision Urine Cytology AI Solution (PUCAS)	2,641 images of urine cytology	2,335 images of urine cytology (retrospective validation cohort) and 400 images of urine cytology (prospective validation cohort)	The sensitivity of PUCAS ranged from 0.922 to 1.000 in retrospective validation cohorts and was 0.896 (0.837-0.939) in prospective validation cohort. PUCAS reduced the use of endoscopes by 57.5% with a negative predictive value of 96.4%.	(38)

AI, artificial intelligence; PUCAS, Precision Urine Cytology AI Solution; CI, confidence interval; DL, deep learning; HGUC, high-grade urothelial carcinoma.

The most readily available and widely used urine biomarker is urine cytology, but urine cytology lacks sensitivity. Combining urine cytology with AI can significantly improve the accuracy of digitized urine cytology slide analysis. Levy et al. (36) reported on AutoParis-X (AP-X), a DL tool that facilitates rapid semi-autonomous examination of urine cytology specimens. AP-X iterated previous algorithms for urine cytology assessment to address many of the remaining complexities. It has been validated on a large scale and has improved the sensitivity of urine cytology. Lebret et al. (17) evaluated the diagnostic performance of the VisioCyt test in terms of sensitivity by urine cytology. The *in-vitro* diagnostic medical device VisioCyt uses whole-section digitization and AI algorithms to identify cancer cells. The results showed a significant increase in the overall sensitivity of the VisioCyt test for detecting BC. According to their study, VisioCyt is reproducible and reliable. Tsuji et al. (37) developed an automated AI system that analyzed 536 consecutive urine cytology slides from 382 patients. AI had 63% sensitivity for high-grade urothelial carcinoma (HGUC) histological prediction, superior to a pathologist’s cytology. The AI predicted histological HGUC from digitized urine cytology slides with great sensitivity and maintained specificity and accuracy (39). Wu et al. (38) conducted a large multi-center cohort study to develop and validate an AI diagnostic system, which was termed PUCAS for urine cytology. PUCAS consists of three stages: patch extraction, feature extraction, and classification diagnosis. It may improve the sensitivity of urine cytology and avoid unnecessary endoscopy.

The significance of AI in urinary cytology diagnosis of BC not only improves the sensitivity and specificity of diagnosis, the AI software system VisioCyt assay offers a significant increase in sensitivity compared to conventional cytological tests, but also by analyzing urine samples for specific markers or changes in cell morphology, achieves non-invasive detection and reduces the pain of patients. Katims et al. (40) found in a retrospective study that genome sequencing was successful in 45 of 48 patient samples with urine cytology, and all patients with negative urine cytology and low-grade tissue had successful cytological sequencing. Chauhan et al. (41) found that DNA minimal residual disease detection for radical cystectomy for bladder cancer was significantly associated with pathological response through a deep sequencing urothelial cancer personalized analysis study, which may be helpful for patients opting for bladder preservation therapy. In the future, it is expected that genomic sequencing technology will be used to obtain genetic information of urinary cells and combined with AI technology to quickly and accurately analyze a large amount of genomic data to analyze whether there are any genetic variants related to bladder cancer, etc.

### 2.6 Molecular biology

AI models developed based on molecular biology information also have multiple uses in diagnosing and treating BC; one of the applications is to use ML analysis of BC molecular markers related to metabolism. Shao et al. (42) profiled metabolite profiles of 87 samples from BC patients and 65 samples from hernia patients and built an ML model, decision trees, based on the metabolomic profiles and six marker candidates. Further studies by Lee et al. (43) demonstrated that the use of surface-enhanced Raman spectroscopy (SERS) combined with ML algorithms to analyze nano-biomarkers in a single drop of urine has the potential to improve the accuracy of early BC diagnostics. In their study, the combination of SERS and ML achieved an accuracy greater than or equal to 99.6% in diagnosing both early- and polyp-stage bladder tumors in a rat model.

### 2.7 The staging of bladder cancer

NMIBC frequently recurs or progresses with five-year rates of 31-78% and 1-45% (44). It is prone to lymphatic metastasis and is less sensitive to chemotherapy, seriously threatening the health of the nation. Through AI technology, precise staging of BC will be achieved, thereby promoting individualized and precise treatment for patients. This can significantly reduce the workload of clinicians, improve the speed and accuracy of diagnosis, make up for the unbalanced distribution of medical resources, promote the standardization and homogeneity of BC diagnosis and treatment, and solve the problem of low-level diagnosis and treatment of BC in grassroots hospitals.

Wu et al. (45) established a lymph node metastasis diagnostic model (LNMDM) based on whole-slide images. A total of 1,012 BC patients who received radical cystectomy and pelvic lymph node (LN) dissection from five hospitals between January 1, 2013, and December 31, 2021, were included. These patients had complete LN section images available for model development. The results showed that the diagnostic accuracy of this model was 97.8% - 99.8%. In the validation sets of the 5 hospitals, the AUC for accurate diagnosis by LNMDM ranged from 0.978 (95% CI 0.960 - 0.996) to 0.998 (95% CI 0.996 - 1.000). Furthermore, the diagnostic sensitivity of this model significantly exceeded that of both junior and senior pathologists. The AI assistance enhanced the diagnostic sensitivity of both junior and senior pathologists. In conclusion, LNMDM shows excellent potential for clinical application in improving the accuracy and efficiency of the work of pathologists. Qureshi et al. (46) hypothesized that MRI/RNA-seq-based radio genomics and AI can stage BC more accurately. A total of 40 MRI and matched formalin-fixed paraffin-embedded (FFPE) tissues were available for their analysis, among which 28 sets were used for training analysis and 12 sets for validation. Overall, the model’s average sensitivity, specificity, and accuracy in differentiating intravesical cancer from extravesical cancer were 94%, 88%, and 92%, respectively. Compared with the genetics-based and radiomics-based models alone, the proposed model improved by 17%, 33%, and 25%, respectively, on the three substrates, and by 17%, 16% and 17% respectively, which meant that it could provide insights into the discriminative features for more accurate staging of BC. Yin et al. (47)provided an ML-enabled, feature-centric, and interpretable diagnostic system to facilitate the precise staging of non-invasive Ta and superficially invasive T1 diseases. 1177 hematoxylin-and eosin-stained (H&E-stained) images of BC tissues were collected by Yin et al., including 460 non-invasive (Ta stage) and 717 invasive (T1 stage) tumors. The features extracted from the images were analyzed using ML methods. By reducing the feature set, they successfully distinguished 1177 Ta or T1 images through six supervised learning methods, with an accuracy rate of 91-96% (47). In contrast, the CNN model that automatically extracts features from images yielded an accuracy rate of 84%. This indicates that the feature extraction driven by domain knowledge is superior to the automatic feature extraction based on CNN.

The application of artificial intelligence in the staging of bladder cancer can improve the staging accuracy while maintaining objectivity and being fast and efficient. Currently, some studies are attempting to use artificial intelligence technologies, such as deep learning algorithms, to analyze the imaging images of bladder cancer (such as CT, MRI, etc.) to achieve more precise staging. Some studies have shown that artificial intelligence has a certain potential to stage bladder cancer and can assist doctors in making decisions. However, problems such as large data requirements, complex models, and insufficient clinical validation still need to be solved. In the future, optimizing the data and carrying out multi-modal fusion to promote its clinical application is necessary.

### 2.8 Histopathology

Within the field of BC histopathology, the application of AI has benefited from the development of digital pathology techniques, mainly using digitized whole section images (WSIs) and immunohistochemistry (IHC) images. Based on these histopathologic images and additional clinical information, we can use various algorithms to make more reliable choices in many dimensions of the clinical diagnosis and management of BC, which include differential diagnosis, grading classification, molecular staging, and prognosis.

Aided by AI models, trained with the WSIs of diagnosed patients, physicians can make a fast and accurate preliminary differential diagnosis of BC in conditions where medical resources are limited. Jansen et al. (48) trained a U-Net segmentation AI based on the DL technique by scanning 328 tumor specimens from three different centers, which can accurately distinguish uroepithelial carcinoma from atypical uroepithelial lesions. Chen et al. (49) constructed an ML-based automated diagnostic model to distinguish normal bladder tissue from BC, with an AUC value of 94.1% in the external validation cohorts. In addition, the team’s model could also discriminate between BC and glandular cystitis, with an AUC value of 93.4% in the patient cohort from Shanghai General Hospital. Using these models can eliminate the variations caused by subjective factors to a certain extent and obtain more accurate results. However, many of these models are limited by the differences in how slides are produced in clinical settings across hospitals, and their performance in clinical simulations is generally inferior to that in training cohorts.

Most AI models used for BC grading are based on H&E-stained sections. Noorbakhsh et al. (50) used DL to analyze the Cancer Genome Atlas (TCGA) database of 27,815 H&E-stained WSIs of different types of cancer. They analyzed them with an accuracy of over 98% for BC classification. In addition, the U-Net segmentation neural network AI trained by Jansen et al. (48) enables automatic detection of NMIBC grading. Pan et al. (51) reported a pathological AI diagnostic model (PAIDM) for BC. This PAIDM was developed using a training set based on the DL algorithm ScanNet, which performed well in diagnosing muscle invasion and identifying histologic grade at the patch level and WSI level. Based on the InternImage-B model with 128 M parameters, Ceachi et al. (52) used the UperNet method to design an AI-based automated approach to detect lymphovascular invasion in urothelial carcinoma by analyzing scans of H&E-stained sections. In their study, their model outlined lymphovascular invasion on H&E-stained slides more efficiently than human examiners. Some other models use different histopathologic images, such as IHC images. Using several CNN-based computational methods, Khosravi et al. (53) built a standalone pipeline to efficiently classify different histopathology images and discriminate subtypes of different types of cancers, including BC. The team provided algorithms for bladder biomarker discrimination up to 99% and Score discrimination in the bladder up to 77%. Lee et al. (54) used the AI-powered product from Lunit, Lunit SCOPE PD-L1, to assist pathologists in completing the assessment of PD-L1 expression levels. Their study demonstrated that AI assistance could improve the consistency of combined positive score results in BC between pathologists and might have the potential to assist in developing multicenter telepathology reading. Using 363 H&E-stained WSIs from the TCGA cohort, Woerl et al. (55) developed the DL model mibCNN to predict molecular subtypes (basal-like, tubulointerstitial, P53-like, and all-negative) in MIBC patients. The AUC value of this model reached 85.00%. In addition, the team used class activation map visualization techniques to identify which histopathological features are most relevant to the model to call a certain molecular subtype, which solves the black-box problem to some extent and facilitates the clinical use of such models.

Similarly, Velmahos et al. (56) extracted imaging biomarkers from H&E-stained WSIs computationally. They used 418 genomic profiles and WSIs of BC cases from TCGA to build a CNN to identify tumor-infiltrating lymphocytes (TIL). The percentage of the tissue containing TIL was then used to predict FGFR activation status with a logistic regression model. Their model can identify patients with any FGFR gene aberration using CNN-based TIL percentages and shows high sensitivity and specificity in predicting patients with only FGFR2/FGFR3 mutation. Loeffler et al. (57) trained a DL network to detect FGFR3 mutations on 327 digitized MIBC sections and validated the algorithm in the “Aachen” cohort, with respectively AUC values of 0. 701 and 0.725, outperforming urologic pathologists. But for now, there is a lack of data from a larger, multicenter, muscle-invasive BC cohort to validate the network further. Noorbakhsh et al. (50) also used a CNN model to distinguish between TP53 mutant/wild-type BC with an AUC value of 0.61. Yan et al. (58) proposed a WSI-based mutation prediction framework that could well predict five clinically relevant mutations (ATM, PIK3CA, ERBB2, FGFR3, ERCC2, with a predicted AUC value of 0.83 or more). They utilized the hierarchical deep multi-instance learning method with a two-stage attention mechanism to identify patches that are highly correlated with gene mutations, improving the interpretability of the model. Using the information extracted from histopathology imaging by such AIs, physicians can initially plan a patient’s treatment cost-effectively.

## 3 AI in the treatment of bladder cancer

### 3.1 Radiation therapy

Radiation therapy (RT) is one of the mainstays of BC treatment. RT is used as the primary treatment for BC in patients with MIBC when preferred surgical intervention is not possible (59, 60) or as a palliative treatment option to relieve symptoms and improve quality of life in patients with advanced or metastatic disease (61). RT has undergone tremendous technological advances in the last three decades, among which the development of AI has significantly benefited RT, including imaging, treatment planning, quality assurance, and outcome prediction. The rapid development of DL has dramatically accelerated the research of AI and its application in RT. **Table 3** mainly introduces AI’s contribution in each RT stage.

**Table 3:** The role of artificial intelligence in all stages of radiation therapy.

Stages of radiation therapy	The contribution of artificial intelligence	Reference
Image reconstruction	Reconstruct Low-dose CT images, Accelerated image acquisition and improve the image quality of CBCT	(62–64)
Bladder Segmentation	DL model makes a significant contribution to bladder segmentation	(65–68)
Target sketch	Enable automatic segmentation across multiple imaging modalities and reduce the total time for OAR generation	(69, 70)
RT planning	DL prediction of dose distribution	(71, 72)
Quality assurance	ML can predict the analysis results of IMRT QA and automatically detect errors in the treatment system	(73, 74)
Prognosis of RT	Models based on results from structured and unstructured datasets to predict whether individual RT of patients has achieved tumor local control or normal tissue toxicity	(75–78)

DL, deep learning; ML, machine learning; RT, radiation therapy; QA, quality assurance; CT, Computed Tomography; CBCT, Cone-beam computed tomography; OAR, organ at risk; IMRT, intensity modulated radiation therapy.

#### 3.1.1 Image reconstruction in radiation therapy

For image reconstruction in radiotherapy, DL has been used to reconstruct low-dose CT images with remarkable success (62, 63). Chen et al. (64) demonstrated a low-dose CT reconstruction method that combines a self-encoder and a CNN. This method extracts fixed-sized plaques from paired low-dose and normal-dose CT images. These patches are transferred to the feature space in a fully connected convolutional layer using a rectified linear cell activation function. In this process, image noise is suppressed. In the decoder step, an inverse convolutional layer is used to recover image details from the extracted features. Cone-beam computed tomography (CBCT) images for image-guided radiation therapy (IGRT) used to verify the patient’s position have lower soft-tissue contrast and higher noise ratios than CT images, which affects the accuracy of image alignment (79). The DL neural network also effectively improves the quality of CBCT by correcting its shading artifacts. It has been shown that deep residual convolutional neural networks (DRCNN) can perform scatter correction by learning to compensate for shadowing compensation maps (80, 81). It is also worth mentioning that there is a technique for online adaptive radiation therapy currently being used in practice in the field of RT, whereby image acquisition, treatment planning (modification), and irradiation of treatment beams are done while the patient is lying on the treatment bed. This technique can be used with MRI-equipped and CT (or CBCT)-equipped devices. Since CT value data from the former are critical for dose calculation, techniques for generating virtual CT images from MRI scans using AI have been developed and implemented in a clinical setting (82).

Although DL shows exciting potential for low-dose CT images, some challenges remain. One of the major criticisms is that for image reconstruction of new patients, deep learning-type reconstruction algorithms may introduce image distortion and artifacts that are difficult to distinguish from the actual anatomical features (83). Therefore, it is critical to investigate further the biological basis of imaging features (84).

#### 3.1.2 Bladder segmentation

Currently, manual segmentation of the primary tumor and affected lymph nodes is one of the most time-consuming yet critical tasks for radiation oncologists (85). The accuracy of tumor segmentation can directly affect the results: an incorrect tumor segmentation may result in underdose or overdose. Tumor segmentation is subject to the individual judgment of the physician, which can lead to differences in the quality of the treatment plan with an impact on survival outcomes (86). In pelvic imaging research, bladder segmentation is also called automatic differentiation between the bladder and its surrounding anatomy. It is the first necessary step to develop a computer-aided diagnosis (CAD) system for BC (65). The bladder has a wide range of shape and size changes and low contrast between the bladder wall and surrounding soft tissue. This makes bladder segmentation a challenging task in medical computer vision. Current semi-automatic segmentation tools that incorporate prior knowledge of reference images, such as segmentation atlas, are not widely available due to their high cost and still require much manual input (87). AI has the potential to significantly improve the efficiency, repeatability, and quality of radiotherapy planning by enabling almost or possibly even completely automated segmentation methods (85). Cha et al. (88) developed a CNN model comprising two convolutional layers, two local connection layers, and a fully connected layer to discern patterns both within and outside the bladder in CT urography images. The output of this model is subsequently represented as a bladder diagram based on its findings, achieving a Jaccard index of 0.76 on the test dataset. The Jaccard index quantifies the similarity between two sets by calculating the ratio of their intersection size to that of their union.

In subsequent research, Ma et al. (89) trained an end-to-end U-net model utilizing the same dataset, thereby obviating the need for user-defined regions of interest (ROI) or auxiliary level set methods for post-processing; this resulted in an increase in the Jaccard index value by 0.09 on the test set (from 0.76 to 0.85). In both studies (88, 89), experienced radiologists provided manual contours of CT slices as reference standards. U-net is characterized by its distinctive U-shaped architecture composed of two CNNs: one transforms input images into features while the other processes these features to yield segmented outputs (90). Similar deep learning models have also been employed for bladder segmentation in MRI scans (91, 92).

#### 3.1.3 Radiation therapy target sketch

Segmentation of the target and organs at risk (OARs) is one of the most important and time-consuming steps in preparing, delivering, and evaluating radiation therapy. Automated image segmentation techniques combining degree learning, decision forests, and generative adversarial neural networks have utility in radiation oncology. 3U-Net was developed to enable automated segmentation across multiple imaging modalities by using available training data more efficiently and reporting localized information about the features (69). DL-based automated contouring of organs at risk reduces the total time required to generate acceptable volumes, including manual editing. A study of automated contouring of the bladder and rectum in 15 prostate cancer patients found that a DL approach reduced the time radiation oncologists spent on editing (4.7 minutes) compared to an atlas-based approach (10.2 minutes) and was similar to a manual approach (4.1 minutes) (70).

Inadequately, AI lacks standardization in image segmentation, feature extraction, and outcome evaluation in RT applications (93). The successful clinical integration of artificial intelligence algorithms necessitates rigorous external validation across diverse cohorts and standardization of models and benchmarks. Numerous studies on artificial intelligence conducted thus far, including several cited in this article, have been limited to small cohorts utilizing a single dataset, failing to capture the rich variability inherent in patients and systems worldwide. Areas with limited standardization of definitions include annotation and contour of organs and nonmalignant tissues, RT techniques, nature and timing of tumor recurrence, severity grading of toxicity, and evaluation concepts and metrics for treatment planning, among others (94, 95). The creation of some medical data repositories (such as the Cancer Imaging Archive) to help facilitate data sharing (96) and attempts by professional organizations to standardize radiation oncology ontologies have contributed to the standardization of AI in RT (97, 98). However, more future work is still needed in this area. It is also important to note that early AI studies in radiation oncology often focused on easily measurable outcomes, such as overall survival. Now, AI solutions will shift toward predicting outcomes more directly related to radiotherapy (85), such as local tumor control and radiation-induced toxicity. However, collecting reliable outcome data remains a challenge.

#### 3.1.4 Planning of radiation therapy

Similar to image segmentation, the research focus of AI in RT planning is on planning automation, including beam direction and dose distribution. AI has long been used for RT planning. It has been reported that DL learns CT contours and dose distribution inputs, automatically generates dose distributions by inputting new contours, generates treatment plans comparable to knowledge-based treatment plans, and is applied to RT planning for prostate cancer (71, 72). Thus, it is possible that DL can fully automate planning from segmentation to optimization in BC RT in the future in hours, minutes, or even seconds. Patrik et al. (99) reported early clinical experience with online adaptive radiotherapy in the pelvic region using a novel AI-driven solution for CBCT. In the clinical trial, treated bladder patients showed a median 42% reduction in primary PTVS, demonstrating the potential for AI-driven automated treatment planning to achieve reduced toxicity.

#### 3.1.5 Quality assurance

The next step in planning is quality control (QC) and quality assurance (QA) of the treatment plan. Dose validation is required once the intensity modulated radiation therapy (IMRT) treatment plan is complete. Currently, this is done using scales, films, or multidimensional detectors to make the actual measurements, which can take several hours of staff time (100). Recently, gamma analysis results measured using 2D detectors were investigated using ML to predict the gamma analysis results of IMRT QA (73). It has also been shown that ML can automatically detect errors occurring in the treatment system (74). Using DL for QA is a critical, promising direction for clinical RT. It is worth mentioning that machine quality assurance includes various assessments of the function, accuracy, and precision of the processing machine on a daily, weekly, monthly, or yearly basis. The large amount of data obtained during these evaluations provides the means to develop AI algorithms capable of predicting trends and errors, such as automatically detecting imaging artifacts, etc. (101), which may provide ideas for data collection in AI in the field of RT. There are still many preclinical and clinical studies in radiation oncology to validate the early findings. These initial studies often face challenges due to relatively small sample sizes, which may limit the generalizations and robustness of their conclusions (102). Collaboration and data-sharing programs among research institutions are essential to address this issue. By pooling resources and expertise, researchers can enhance the statistical power of their analyses. In addition, a promising approach using distributed learning techniques is proposed to train predictive models based on aggregated data while ensuring patient privacy. Distributed learning allows multiple institutions to participate in model development without directly sharing sensitive patient information. This approach allows for confidentiality and researchers to access a more diverse data set that reflects diverse demographics and treatment responses. In addition, fostering partnerships between academic institutions, healthcare providers, and technology companies can facilitate innovative data collection and analysis approaches. Such collaborations may improve study designs that integrate real-world evidence with findings from traditional clinical trials. Ultimately, it is possible to accelerate the validation process in early studies by adopting these collaborative strategies and advanced analytical techniques in radiation oncology research. This will significantly enhance our understanding of treatment effects while facilitating advances in personalized medicine based on comprehensive data sets from across the medical community working together.

Currently, AI has been reported less in RT for BC compared to other pelvic tumors such as prostate, rectal, and cervical cancers. Still, AI is playing an increasingly significant role in the development of RT. It can be foreseen that AI will play an integral role in RT for BC in the future.

## 4 AI in the prognosis of bladder cancer

Stratifying cancer patients according to their prognosis or treatment response can suggest the best treatment plan, reduce ineffective treatment, and improve overall survival and quality of life through personalized patient management. So far, very few data about BC prognosis. At the beginning of the 21st century, Catto et al. (103) were the first to use machine learning models to predict BC recurrence and progression using clinical and histopathological data. Only recently have AI-based models been used again to predict prognosis in BC (104). The application of artificial intelligence technology to the diagnosis and prognosis of BC can significantly improve the quality of life of patients by preventing unnecessary radical cystectomy, which is the most expensive malignancy to treat in a patient’s lifetime, while reducing the financial burden of patients.

### 4.1 Overall survival rate and the recurrence rate of prediction

Prognosis prediction is an essential part of clinical oncology, as the expected disease path and survival likelihood influence treatment decisions (105). Lucas et al. (106) used a deep learning-based model combining both clinical and histopathological data to predict recurrence. In terms of muscle-invasive bladder cancer, AI may bring more personalization and Patient based treatment. The 5-year survival rate of patients with NMIBC is about 90% (66). Patients with MIBC have dramatically decreased 5-year survival rates due to tumor invasion into different levels of the bladder. Patients with the same tumor stage can also have significant differences in survival outcomes due to individual differences. Therefore, the development of a model that can accurately estimate the disease-specific death risk of individual patients will help oncologists develop appropriate treatment strategies to assess that cystectomy is a surgery with high mortality. Although there are cases that have been treated by endoscopic resection or without intravesical intervention, resection is still the first intervention for bladder cancer patients with a high risk of progression. However, more than 60% of patients develop at least one complication within 90 days after cystectomy and have poor tumor control long-term after surgery (66). Even with negative surgical margins, the 5-year overall survival rate for patients who did not receive neoadjuvant chemotherapy was only about 47%. In one study, Song et al. (107) used a machine learning model constructed from a dataset of 1228 patients to assess the 10-year survival of BC. The author in the model design, in addition to the tumor grade, because of its lack of reliability between the observers. The final model had an area under the AUC of 0.77 and an F1 score of 0.78, which showed that the final model could predict 10-year BC survival.

Approximately 30% of patients with NMIBC and MIBC relapse after cystectomy (44). Therefore, accurate and reliable prediction of tumor recurrence is essential for patients with NMIBC and MIBC. Xu et al. (108) used a preoperative MRI dataset of 71 BC patients to build a nonlinear support vector machine (SVM) classification model to identify patients at risk of recurrence within two years after surgery. The authors extracted 1872 image features from each patient’s T2W, DW, apparent diffusion coefficient, and dynamic contrast enhancement maps. They used a recursive feature elimination scheme to select the optimal subset of features for the classification task at hand. The final SVM model included 32 radiomics features, and the AUC was 0.8593 and 0.8216 in training and validation, respectively (108). In a distinct study, Hasnain et al. (109) utilized one of the largest single-institution cystectomy datasets collected by the University of Southern California Institute of Urology over 45 years (1971–2016) to create fundamental classifiers for predicting recurrence at 1, 3, and 5 years. This dataset includes comprehensive information on 3499 patients, encompassing demographic characteristics and comorbidities before cystectomy, tumor markers and clinical diagnostic data obtained during the surgical procedure. For each predictive task in developing a meta-classifier across all timeframes (1, 3, and 5 years), both sensitivity and specificity scores exceeded 0.7.It is worth mentioning that Sun et al. (110) developed a method based on clinical, radiomics, and deep learning descriptors to improve survival prediction. The authors analyzed the clinical data through Nomogram, the image data using radiological and deep learning models, and the features of the three descriptors for each condition were put into the backpropagation neural network for survival prediction. The results showed that clinical, radiomics, and deep learning have great potential in predicting the survival of BC patients after cystectomy. Sun et al. (111) also combined AI and large language models (LLMs) to extract clinical information and improve image analysis. They evaluated the accuracy of LLM retrieval by a multimodal prediction model based on clinical, radiological, and deep learning descriptors developed by the authors. The results showed that the performance of a model using clinical descriptors extracted from LLM as nomogram input for predicting 5-year bladder cancer survival after cystectomy was comparable to that of a model using manually extracted clinical descriptors.

### 4.2 The evaluation of response to therapy

DL of neural networks and the development of the group of radiology imaging technology of the latest improvement can be used to predict response to therapy (such as new adjuvant chemotherapy). Cha et al. (112, 113) investigated the contribution of post-neoadjuvant chemotherapy CT to assessing BC response using deep learning convolutional neural networks. Kong et al. (114) used web-based machine learning to evaluate different biological markers in a bladder organ model to predict the efficacy of anticancer drugs and ultimately found that the best response to drugs was in a manner in which amino acid synthesis and performance varied with each other. More recently, Manitz et al. (115)used ML in the JAVELIN Bladder 100 phase 3 trial for additional exploratory purposes in maintenance and prospective studies, with final analyses identifying potential prognostic and predictive factors for avelumab 1 L maintenance therapy in BC patients. In the evaluation of neoadjuvant chemotherapy response to bladder cancer, the practice of AI is still progressing.

In addition, computerized decision support systems (CDSS-T) may improve diagnostic accuracy. Cha et al. (113) evaluated pre - and post-chemotherapy CTU scans of 123 patients (157 pre - and post-treatment cancer pairs) for bladder cancer treatment response without CDSS-T and with CDSS-T and showed that CDSS-T improved CT performance in identifying complete response of muscle-invasive bladder cancer to neoadjuvant chemotherapy. Another study by Sun et al. (116)also showed that CDSS-T can improve the diagnostic accuracy of CT urography in assessing the response to treatment of bladder cancer, especially in multi-specialty and multi-institutional Settings, resulting in more consistent diagnostic performance among physicians.

In the domain of bioinformatics, gene expression analysis is investigated as a tool for predicting treatment responses, including neoadjuvant chemotherapy and immunotherapy, among others (117). Kong et al. (114) utilized the pharmacogenomic data of 3D colorectal cancer (n = 19) and bladder cancer (n = 9) organoids from the STRING database to construct a protein-protein interaction (PPI) network. They developed a machine-learning framework to identify biomarkers to predict patients’ responses to drugs. This research addresses the issues confronted by preclinical models and computational methods in predicting cancer drug responses, which typically stem from the insufficient validity of *in vitro* models and the data availability constraints of training algorithms. The foundation of this approach lies in the fact that genes associated with similar phenotypic outcomes usually exhibit close connections in the PPI network, and through extended analysis, biomarkers of specific drug responses might also have close associations in the interaction network (118). The study’s outcomes emphasize the necessity of appropriate *in vitro* drug screening models, network analysis’s benefits in identifying strong predictors of responses, and the capacity of network-based ML models to effectively predict which patients may or may not respond to specific drug treatments (119).

### 4.3 Pathological evaluation of prognosis

AI models can improve predictive accuracy by combining digital images and other clinical information. In 2002, Spyridonos et al. (120) trained a neural network-based computerized diagnostic prognostic system by combining histologic (subjective) features assessed by pathologists and automatically extracted nuclear features, achieving a prognostic assessment accuracy of 72.8%. In 2020, Harmon et al. (121) trained a DL model that could predict LN metastasis based only on digitized pathologic images of primary BC, with an AUC value of 0. 866, which outperformed the clinicopathologic multivariate logistic regression model with clinical pathology features. In 2022, Lucas et al. (106) found that a DL-based model that combines digital histopathology sections with clinical data improved the prognostic accuracy of the patient’s prognosis, compared with models that use only clinical or image data. These studies indicated that data from a DL-based model trained with digital images and other clinical information may improve the accuracy of recurrence prediction.

Also in 2022, Tokuyama et al. (122) successfully constructed a system to predict the recurrence of NMIBC with high prognostic accuracy by capturing only the nuclei of transurethral resection of bladder tumors (TURBT) samples to eliminate the effect of sampling conditions. In 2024, Van Rijthoven et al. (123) proposed a method based on the multiresolution DL model HookNet-TLS for automated, unbiased quantification of tertiary lymphoid structures (TLS) and identification of germinal centers in routine hematoxylin-and-eosin-stained digital pathology sections. However, expansion of the BLCA cohort is needed to validate the prognostic relevance of HookNet-TLS predicted regions.

In addition, some researchers have gone further by departing from the classification criteria proposed by the World Health Organization for prognostic purposes, using AI to create new classification criteria, and utilizing the WSIs to accomplish more accurate prognostic speculation. Chen et al. (49) developed an integrated nomogram based on their model’s risk scores and clinicopathological features with higher predictive accuracy than current tumor staging/grading systems, with AUC values of 77.7%, 83.8%, and 81.3%, respectively.

### 4.4 Genetics

BC is one of the most common diseases of the urinary system. Changes in multiple genes and molecular pathways are related to its occurrence. P53 is a tumor suppressor gene that plays an important role in the human body. Its mutation will lead to the disorder of cell apoptosis and DNA damage repair to promote the growth and transfer of bladder cancer cells. Abnormal expression of the TERT gene is also closely related to the occurrence of bladder cancer. TERT is an enzyme that can keep cells dividing (9). Its expression level in bladder cancer is often high. The research focus includes targeted treatment of it.

Poirion et al. (124) identified two subtype populations in BC patients with significantly different overall survival (OS) by using a DL approach. In the aggressive subtype, KRT6 and KRT14, which have already been reported as markers of basal subtype, were upregulated. DLK1, TRH, and DEFB103B were the top genes upregulated in this aggressive subtype. When compared with the less aggressive subtype, the PI3K-Akt pathway was one of the most activated pathways in the aggressive subtype (125).

Tumor microenvironment (TME) plays a crucial role in cancer progression, metastasis, and treatment response (126). The role of the TME, particularly tumor-infiltrating B lymphocytes, was assessed using an ML-based computational framework. The signature based on long non-coding RNA of B lymphocytes in the tumor environment can predict the survival outcome of BC patients and correlate with the response to immunotherapy in patients receiving BC anti-programmed death-1 therapy (anti-PD1). It is worth mentioning that using DL to integrate multiple omics data seems to be the BC survival risk stratification of patients with significant progress. However, further efforts and validation are still needed for clinical applicability (127).

### 4.5 Prognosis in molecular biology

Some studies have sought to employ ML to analyze biomolecules to predict patients’ sensitivity to specific drugs. Zhu et al. (128) used the SVM with radial basis function (RBF) kernel to construct a specific drug response in a platinum-based chemotherapy prediction model for 11 cancers, including BC.

In addition, AI is used for BC-related gene expression analysis to obtain patient treatment and prognosis recommendations. Smith et al. (129) developed a gene expression model (GEM) to predict the pathological node status in primary tumor tissue from three independent cohorts of patients who underwent cystectomy and lymphadenectomy for BC. This GEM can predict pathologically positive LNs based on a subset of transcripts detected by microarrays to predetermine a patient’s risk of recurrence and aid in selecting patients to receive neoadjuvant chemotherapy. Ciaramella et al. (130) analyzed the expression profiles of specific DNA regions from surgically removed normal and BC tissues and developed a methodology based on ML algorithms for the selection of a predictive signature panel for BC onset. Bartsch et al. (131) used an ML algorithm to identify the genes in a molecular signature in patients with non-muscle invasive urothelial carcinoma at initial presentation that was most predictive of recurrence. In their study, a singular 3-gene rule was constructed that predicted recurrence with 80% sensitivity and 90% specificity in the training set and 71% and 67% in the test set, respectively. Wu et al. (132) proposed a genomic-clinicopathologic nomogram for the preoperative prediction of LN metastasis in BC based on a logistic regression algorithm in ML. Combining a five-mRNA-based classifier with three clinicopathological risk factors, the nomogram can predict LN metastasis in BC patients with an AUC value of 0.7867. Fu et al. (133) subtyped BC patients based on the expression patterns of endothelial cell (EC) -related genes and constructed a diagnostic signature and an endothelial cell prognostic index via ML algorithms, which are useful for diagnosing BC patients, predicting the prognosis of BC and evaluating drug sensitivity. In 2024, Li et al. (134) established a novel FRG index (FRGI) and CD8-FRG isoforms by screening 54 essential fibroblast-associated genes (FRG) associated with BC from a large-scale ML-based scRNA sequence dataset. Although there is a lack of clinical phase 3 randomized controlled trials to validate patients’ prognosis and treatment response based on defined FRG subtypes, their FRGI and corresponding subtypes have performed well in multiple cohorts representing bladder cancer. They can accurately predict clinical outcomes and immunotherapy response of BLCA after surgery.

In addition to these direct clinical applications, AI algorithms are also being used to study driver genes and mutations in BC. They are expected to lead to the invention of new treatments in the future. Ellrott et al. (135) proposed an approach for mutation calling of tumor exomes using multiple genomic pipelines, including ML algorithms. They reported that urothelial bladder carcinoma had greater than 90% of the original variants rediscovered. Subsequently, Bailey et al. (136) analyzed 9,423 tumor exomes from the PanCancer Atlas using PanSoftware and made novel predictions, including GNA13 in BC. Chen et al. (137) identified SMAD6 as a hub gene contributing to the differences between two novel subtypes of BC with completely different biological characteristics, including immune microenvironment and drug sensitivity with ML classifiers, and further investigated the role of SMAD6 in BC in the single-cell transcriptome data. This study is expected to improve the future understanding of the prognosis and drug susceptibility of BC. Through a series of studies that performed prognostic diagnosis and prediction of BC patients, the results of the studies were overall promising. Still, major challenges remained to be addressed before these models could be integrated into clinical workflows. Poor ubiquity is probably the biggest obstacle to the clinical translation of AI. This problem is manifested in the fact that AI algorithms perform below par when evaluated on data collected by institutions that are different from those that provide training data. This requires promoting data sharing among medical institutions and establishing a unified medical data platform. Through data sharing, the training data of AI systems can be enriched to improve their accuracy and generalization ability.

### 4.6 The prognosis of radiotherapy

In the tumor treatment, patients with AI to predict individual RT have been optimized for tumor local control or normal tissue toxicity (75, 76). Models based on structured and unstructured data set results prediction have gradually been applied. Luo et al. (77) observed that for structured data, such as clinical and dosimetric data, random forests and gradient-boosted machines produced the most accurate results, while linear regression also performed well due to the relatively small number of high-dimensional data points available in radiation oncology studies. Deep neural networks have a role in generating predictions for unstructured data, including the use of imaging or medical records, but because models contain millions of parameters, overfitting of small data sets remains a concern, and accuracy can continue to improve with more advanced algorithms and data sets (78). On independent data sets strict test, the best is for prospective patients with an accumulation of rigorous testing, the value of this to prove that the AI in outcome prediction is very important.

CAD-AI refers to the application of CAD development and the use of ML - and DL-based methods. Many relevant studies have been published to date. However, most models in these studies still need to be prepared for clinical deployment. The most important is to ensure that clinical decision support tools are adequately trained and rigorously validated for generalizability and robustness before they are used in clinical patient care. Recommendations from the American Association of Physicists in Medicine (AAPM) for practices and standards development for the development and performance evaluation of computer-aided decision support systems may improve their generalizability and reliability and accelerate the adoption of CAD-AI systems to support clinical decision-making (138).

The application of AI to outcome prediction in RT also faces another major bottleneck, namely, the data used for training and testing. Unlike tasks such as segmentation, image synthesis, or dose prediction, a powerful network only needs to train less than 100 patients, and outcome prediction often requires a larger amount of patient data for AI training. It is worth mentioning that the data needed for outcome prediction are generally more difficult to obtain. In AI research, the time and effort to acquire data are often significantly greater than model construction and training. However, with the high quality and quantity of patient data and the opacity of deep learning neural networks, the robustness and generalization of many models used for outcome prediction can be further tested and improved. This means that there is an urgent need to establish and enhance public databases in related fields, such as the Cancer Genome Atlas and the Cancer Imaging Archive (96, 139), which have profoundly contributed to the progress in the fields related to omics and medical imaging.

## 5 Ethics and privacy in artificial intelligence

Often heralded as a potential disruptor, AI has transformed the traditional process of diagnosing and treating disease and is used in many healthcare applications (140). However, as AI continues to evolve and become increasingly integrated into healthcare systems, a range of key moral and ethical issues are gradually being considered (141). These implications relate to privacy, data security, accountability, transparency, fairness, and the preservation of human autonomy, and there is an urgent need to find solutions to realize the potential of AI in clinical care (142).

Among these moral and ethical issues, the most sensitive aspect of patient data privacy is hardly addressed at present. Models such as DL and ML of AI must have large datasets to appropriately categorize and make predictions for accomplishing various tasks. However, due to the confidentiality of patient data, AI cannot readily use the relevant data required (143). Similarly, any leakage of patient privacy-related data could have serious consequences for patient trust and data integrity (142). Given the growing public concern about data security, algorithmic bias, and ethical considerations in using AI, a more thorough exploration of these issues is essential.

It has been suggested that user trust and effective interaction with AI systems can be achieved by increasing interpretability (i.e., the ability of an AI system to explain its decisions, processes, and behaviors in a way that is understandable to humans) (144). Transparent decision-making processes are critical in healthcare AI, where patients and healthcare providers must understand the rationale behind AI-driven recommendations, fostering trust and accountability. A recent survey showed that patients were more likely than doctors to believe doctors should be held responsible for errors caused by medical AI, a view held by majorities in both groups (145). So, there is also research that points to the need for clear accountability for the actions of AI systems (146). Determining who is responsible for errors or adverse events is critical for ethical use and fostering trust. Additionally, healthcare organizations using AI technologies must adopt responsible practices for collecting, storing, and utilizing patient data. This includes robust data anonymization techniques, encryption, and secure data-sharing protocols to protect patient information (147).

Currently, research on addressing AI’s ethical and moral limitations remains in the theoretical stage, and many new ideas are beginning to be proposed. We need to be guided by existing ethical frameworks and look for healthcare-specific, adaptable guidelines to navigate this changing landscape created by AI’s rapid development. Healthcare systems, technologists, policymakers, and healthcare professionals must keep ethics and morality at the forefront to ensure that AI realizes its vast potential in healthcare.

## 6 Discussion

With the continuous progress of AI, the diagnosis and treatment of BC have ushered in a new change, and precision oncology has made significant breakthroughs.AI has made significant progress in the diagnosis of bladder cancer, especially in various aspects such as cystoscopy, imaging histology, urocytology, and molecular biology. Through AI technology, accurate staging of bladder cancer can be realized. In addition, deep learning models are also applicable to bladder segmentation in MRI scans. The application of these diverse algorithms enables clinicians to make more reliable choices when performing multiple clinical dimensions such as differential diagnosis, grading and classification, molecular staging, and prognostic assessment of bladder cancer. AI also plays a role in treatment and prognosis. For example, in radiation therapy, low-dose CT images have been successfully reconstructed by deep learning techniques with remarkable results. Recently improved radiological imaging techniques based on deep learning and neural networks have also been used to predict patient response to treatment.

Although AI has been successfully applied in several fields, it still faces significant challenges. First, at the data level, most studies on the application of AI in bladder cancer have limitations. Some studies have small sample sizes due to difficulties in accessing medical data. Improving the robustness of AI models, even if they remain stable and reliable performance in the face of uncertainties such as noise, outliers, and changes in data distribution, requires a large amount of patient data to avoid the phenomenon of overfitting (i.e., algorithms that perform well on the training set but poorly on a new dataset) and to develop accurate and effective models. Therefore, the establishment and improvement of open medical databases and resource sharing are of great significance in solving the data access problem. Different healthcare organizations actively contribute to public databases, where the burden of data is shared by the community, and the impact of data is multiplied. At the same time, it should not be overlooked that most studies have yet to improve the completeness and homogeneity of the patient monitoring process to comprehensively assess treatment effects. This implies that researchers need to strengthen the tracking coverage during patient monitoring and the standardization of data recording and collection at each step. In addition, special attention should be paid to data preprocessing of image datasets within the field of imaging research, such as removing artifacts, which will help improve the accuracy of image analysis results.

Standardization is another important challenge that needs to be overcome. Currently, the evaluation criteria for AI in clinical applications have yet to be standardized, with image segmentation, feature extraction, and outcome assessment in radiation oncology being particularly prominent. This phenomenon partly stems from the fact that relevant studies have been limited to datasets from a single small cohort. Therefore, current or future clinical studies should focus on how to effectively utilize AI in different clinical settings to further validate and evaluate the applicability of the model in different patient populations. As mentioned earlier, open access to medical databases can provide the necessary resources to address standardization issues. In addition, enhancing the model’s ability to adaptively learn during clinical implementation, continuously monitoring its performance and updating it with new data would be beneficial to enhance its utility in various scenarios in different industries.

Ethical and privacy issues are equally important barriers that limit the full potential of AI. Issues related to patient privacy protection, informed consent requirements, data security safeguards, and the responsibility of healthcare professionals for validating and interpreting AI-generated results are important ethical issues that must be carefully considered prior to the widespread implementation of AI. Interpretability and transparency are important factors in ensuring that AI systems can be trusted and used ethically, as they allow people to understand and evaluate the decision-making process while ensuring responsible and beneficial use of these systems. In addition, laws and regulations governing the use of AI need to be improved to ensure that accountability mechanisms are successfully implemented and that the identity of those responsible for adverse events is clarified in order to enhance patient trust.

Finally, scalability is key to realizing the clinical applicability of AI algorithms, data, and models in the field of bladder cancer. Current AI-based diagnostic tools are not yet optimized for the scale, speed, and complexity required for chylomicron cancer evaluation, which is a major obstacle to their widespread use in clinical practice. At the same time, high-complexity machine learning algorithms require considerable economic cost and computational power to run. To date, implementing these technologies in large-scale healthcare systems has been challenging and requires a robust infrastructure to support their widespread adoption.

Despite the limitations, it is foreseeable that as technology develops and research progresses, AI will show great potential in clinical applications such as chyloma diagnosis and treatment.

## 7 Summary

This article reviews the application of AI in the clinical diagnosis, treatment, and prognosis of BC. Artificial intelligence and its subsets, such as machine learning, deep learning, and artificial neural networks, have become powerful tools in the field of bladder cancer research and treatment, revolutionizing the current and future landscape in terms of early detection, accurate diagnosis, and personalized treatment, as well as predicting disease progression. AI can help clinicians accomplish many clinical practices by analyzing large amounts of data. However, there are still challenges to overcome, such as data acquisition, standardization development, and ethical considerations. However, with continuous research and technological progress, these problems will be solved, and AI will play its due potential in the BC field.
